# Ellagic acid attenuates interleukin-1β-induced oxidative stress and exerts protective effects on chondrocytes through the Kelch-like ECH-associated protein 1 (Keap1)/ Nuclear factor erythroid 2-related factor 2 (Nrf2) pathway

**DOI:** 10.1080/21655979.2022.2059995

**Published:** 2022-04-04

**Authors:** Wenrun Zhu, Han Tang, Juncheng Li, Rui Miranda Guedes, Lu Cao, Changan Guo

**Affiliations:** a Department of Orthopedic Surgery, Zhongshan Hospital, Fudan University, Shanghai, China; b LABIOMEP, UMAI-INEGI, Faculty of Engineering of the University of Porto, Porto, Portugal

**Keywords:** Ellagic acid, osteoarthritis, oxidative stress, chondrocytes, nrf2

## Abstract

Osteoarthritis (OA) is the most prevalent type of degenerative joint disease, and its pathological progression is highly associated with oxidative stress. Natural antioxidants can attenuate oxidative stress and chondrocyte injury, suggesting that antioxidants have potential applications in the management of OA. Ellagic acid (EA), a natural polyphenol derived from fruits or nuts, exerts antioxidant and anti-inflammatory effects in diseases related to oxidative stress. Herein, we investigated the effects of EA on interleukin-1β (IL-1β)-induced oxidative stress and degeneration in C28/I2 human chondrocytes. EA efficiently suppressed IL-1β-induced oxidative stress and ameliorated oxidative stress-induced dysfunction of chondrocytes, as indicated by the promotion of cartilage matrix secretion. Moreover, EA remarkably suppressed cell apoptosis and senescence, and reduced the expression of proinflammatory factors and metalloproteinases, suggesting that EA could alleviate chondrocyte injury under oxidative stress. Mechanistically, EA upregulated the expression of nuclear factor erythroid 2-related factor 2 (Nrf2) as well as its downstream targets NADPH quinone oxidoreductase 1 and heme oxygenase-1. ML385, a specific Keap1/Nrf2 pathway inhibitor, blocked the antioxidant and chondroprotective effects of EA. Our findings demonstrated that EA could attenuate oxidative stress and exert protective effects on chondrocytes by upregulating the Keap1/Nrf2 signaling pathway.

## Graphical abstract

**Figure:**
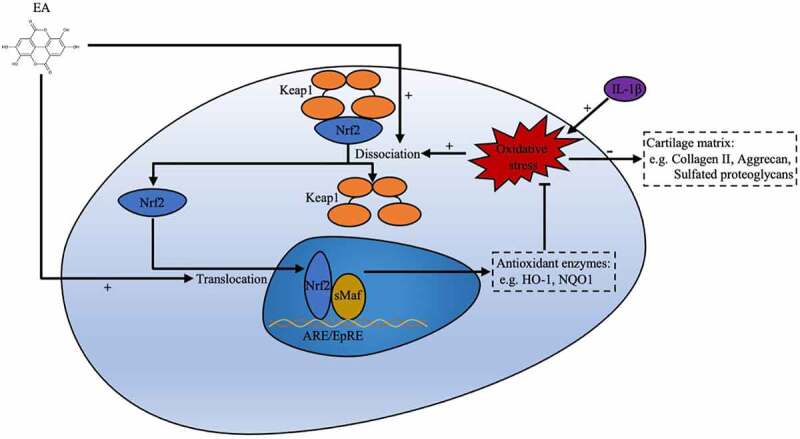

## Introduction

Osteoarthritis (OA) is an age-related degenerative joint disease with a high incidence rate [1], it damages the entire joint structure, including the articular cartilage, the synovium, and the subchondral bone [2]. Most patients with advanced OA eventually require joint replacement, which entails a significant social and economic burden [3]. Despite the improvement in treatments used to relieve symptoms, the nonoperative management of OA remains challenging, especially with respect to effective therapy capable of preventing the pathological progression of OA.

Recent studies have suggested that oxidative stress plays a crucial role in the progression of OA [4]. Progression is associated with oxidative-induced cartilage breakdown and an imbalance between anabolic and catabolic factors of chondrocytes [5]. Elevated oxidative stress, with an increase in the generation of reactive oxygen species (ROS) from chondrocytes, can eventually lead to an inflammatory response [6], cellular senescence [7], and apoptosis [8]. Previous studies have reported that excessive oxidative stress and ROS production are detected in patients with OA [9,10]. Furthermore, recent research has suggested that excessive ROS generation occurs during the development of OA, resulting in increased inflammation [11,12]. Therefore, suppression of oxidative stress and excessive ROS production is vital for protecting the regenerated cartilage obtained by tissue engineering for the restoration of OA cartilage defects.

Nuclear factor erythroid 2-related factor 2 (Nrf2), a key regulator that maintains cellular redox homeostasis, is an oxidative stress-sensitive transcription factor [13]. Under physiological conditions, its combination with Kelch-like ECH-associated protein 1 (Keap1) restricts the localization of Nrf2 to the cytoplasm, where the ubiquitin proteasome system constantly degrades Nrf2 [14]. Under oxidative stress conditions, Nrf2 can dissociate from its repressor Keap1, translocate into the nucleus, and bind to the antioxidant response element (ARE) to upregulate the expression of various antioxidant genes, including heme oxygenase-1 (HO-1) and NADPH quinone oxidoreductase 1 (NQO1) [13]. Nrf2/ARE pathway agonists have provided beneficial effects in experimental models of chronic diseases, such as cardiac disease, diabetes, and neurodegenerative diseases [15]. Furthermore, a recent study has reported that glycyrrhizic acid alleviates acute lung injury in neonatal rats through the Keap1/Nrf2 pathway [16]. In OA, Nrf2 activation relieves inflammatory responses in cartilage [17]. Furthermore, linagliptin restores the low expression of ECM-related genes partially through the Nrf2/SOX9 axis in C28/I2 human chondrocytes [18]. Thus, regulation of Nrf2 activity might have a therapeutic effect in preventing the pathological progression of OA and may contribute to the maintenance of the regenerated cartilage.

**Figure 1.**
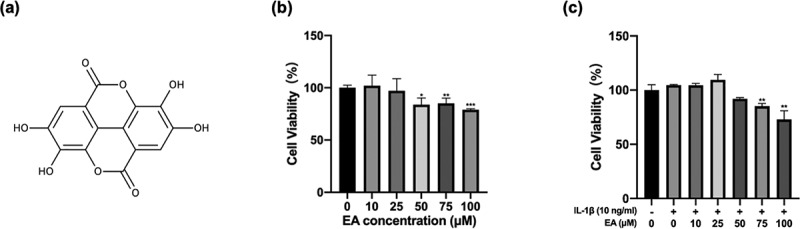
Effects of ellagic acid (EA) on cell viability. (a) EA chemical structure. C28 chondrocytes were exposed to EA (0 μM, 10 μM, 25 μM, 50 μM, 75 μM, 100 μM) alone (b) or with interleukin (IL)-1β (10 ng/mL) (c) for 24 h, and cell viability was measured using the Cell Counting Kit-8 (CCK8) assay. *N* = 3. The values are expressed as the mean ± standard deviation. *p < 0.05, **p < 0.01, and ***p < 0.001 vs. control group.

EA (Figure 1(a)), a natural bioactive agent derived from the peel of fruits and nuts, possesses multiple biological properties, including anti-inflammatory [19], antidiabetic [20], and antioxidant [21] activities. In a recent study, EA alleviated oxidative stress and insulin resistance in HepG2 cells exposed to high glucose through the Keap1/Nrf2 signaling pathway [22]. However, it remains unclear whether EA can ameliorate oxidative stress of chondrocytes and exert a chondroprotective ability through the Keap1/Nrf2 pathway.

Herein, we predicted that EA could exert antioxidant and chondroprotective effects on chondrocytes via the Keap1/Nrf2 pathway. We investigated the antioxidant and protective effects of EA on interleukin (IL)-1β-treated C28/I2 human chondrocytes and clarified the underlying mechanism. Our findings might lay a theoretical foundation for OA treatment with natural antioxidants.

## Materials and methods

### Cell expansion

C28/I2 human chondrocytes were acquired from the cell bank of the Chinese Academy of Sciences (Shanghai, China). Chondrocytes were cultured in Dulbecco’s modified Eagle medium: nutrient mixture F-12 (DMEM/F-12) (Gibco, Carlsbad, CA, USA) with 10% fetal bovine serum (FBS) (Gibco), 1% penicillin, and streptomycin (Sigma-Aldrich, St. Louis, MO, USA) at 37°C with 5% CO_2_. The medium was replaced every 3 days.

### Cell viability assay

C28/I2 chondrocytes were grown in 96-well plates at a density of 5 × 10^3^ cells/well. Cells were then cultured with different concentrations of EA (Rhawn Chemicals, Shanghai, China) with or without IL-1β (10 ng/mL) (R&D System, Minneapolis, MN, USA) for 24 h. A Cell Counting Kit-8 (CCK8) (Dojindo, Tokyo, Japan) was used to assess the cell viability of chondrocytes. Briefly, 100 μL of serum-free DMEM/F-12 mixed with 10 μL of CCK8 regent was added into each well and incubated at 37°C with 5% CO_2_ for 1 h. Thereafter, the absorbance of each well was examined at 450 nm with a microplate reader (Infinite F200, Tecan, Männedorf, Switzerland).

### Intracellular ROS measurement

A fluorescent probe, 2’,7’-dichlorofluorescin diacetate (DCFH-DA) (Sigma-Aldrich), was used to measure intracellular ROS levels of C28/I2 chondrocytes. Chondrocytes were grown in 96-well plates. After a 24-h treatment, each well was washed with phosphate-buffered saline (PBS) (Gibco) twice; then, 1 mL of serum-free DMEM/F-12 mixed with 10 μM DCFH-DA was added, and wells were incubated at 37°C for 25 min in the dark to enable DCFH-DA to enter the cells. The cells were then washed with PBS three times to eliminate extracellular DCFH-DA. ROS levels were detected with a fluorescence microscope (Evos M7000, Thermo Fisher Scientific, Waltham, MA, USA) using a 488 nm excitation wavelength and a 525 nm emission wavelength, and the images were analyzed with Image J software (NIH, Bethesda, MD, USA). The results are displayed as the relative fluorescence intensity [23].

### Malondialdehyde assay

C28/I2 chondrocytes were cultured in six-well plates and collected after a 24-h treatment. RIPA buffer (Beyotime Biotechnology, Shanghai, China) supplemented with phenylmethanesulfonyl fluoride (PMSF) was applied to prepare the cell lysates. The Lipid Peroxidation MDA Assay Kit (Beyotime Biotechnology) was used to quantitatively measure malondialdehyde (MDA) levels, which were detected at an absorbance of 523 nm using a microplate reader. The MDA levels were standardized by protein content, and the protein concentration was assessed using a BCA Protein Assay Kit (Thermo Fisher Scientific) [24].

### Superoxide dismutase quantification

The Total Superoxide Dismutase Assay Kit with WST-8 (Beyotime Biotechnology) was used to measure superoxide dismutase (SOD) activity. C28/I2 chondrocytes were seeded on six-well plates and collected after a 24-h treatment. An SOD sample preparation solution from the kit was applied to prepare cell lysates; 20 μL of cell lysate supernatant and 160 μL of WST-8/enzyme were then added into a 96-well plate. Subsequently, 20 μL of a reaction starter working solution was added into each well. After 30 min of incubation at 37°C, the absorbance of each well was detected at 450 nm using a microplate reader [25].

### Alcian blue staining

A total of 1.5 × 10^5^ C28/I2 chondrocytes were resuspended in 10 μL of DMEM/F-12 supplemented with 10% FBS and seeded as micromasses at the center of each well of a 24-well plate. The chondrocytes were allowed to adhere at 37°C for 1 h, after which 500 μL of control DMEM/F-12, DMEM/F-12 mixed with IL-1β (10 ng/mL) or DMEM/F-12 mixed with IL-1β and EA was added to the wells [26]. After 24 h of treatment, micromasses were stained with Alcian blue dye prepared from Alcian blue 8GX (Sigma-Aldrich), acetic acid solution (Sigma-Aldrich), and double-distilled water.

### Mitochondrial membrane potential assay

Measurement of the mitochondrial membrane potential was conducted with a Mitochondrial Membrane Potential Assay Kit with JC-1 (Beyotime Biotechnology). C28/I2 chondrocytes were grown in 96-well plates. After 24 h of treatment, the chondrocytes were harvested and washed twice using PBS. The chondrocytes were then resuspended in a mixture of 500 μL of JC-1 dye and 500 μL of DMEM/F-12 at 37°C in the dark for 20 min. Subsequently, the chondrocytes were washed with ice-cold staining buffer three times before evaluation with a fluorescence microscope. JC-1 aggregates and displays red fluorescence in cells with high mitochondrial membrane potential, when exposed to a 525 nm excitation wavelength and a 590 nm emission wavelength. In cells possessing low mitochondrial membrane potential, JC-1 exists mostly in a monomer form that emits green fluorescence; this response was detected using a 490 nm excitation wavelength and a 530 nm emission wavelength. The mitochondrial membrane potential was presented as the ratio of red to green fluorescence [27].

### Senescence β-galactosidase staining

β-Galactosidase staining was conducted using a Senescence-Associated β-Galactosidase Staining Kit (Beyotime Biotechnology). After 24 h of treatment, C28/I2 chondrocytes were washed three times using PBS and fixed using 4% paraformaldehyde at room temperature for 15 min. The adhered chondrocytes were then incubated at 37°C overnight in the dark in a working solution containing 0.05 mg/mL 5-bromo-4-choloro-3-indolyl-β-d-galactopyranoside (X-gal) [28].

### Western blotting analysis

Adherent C28/I2 chondrocytes were washed twice by PBS and RIPA buffer containing PMSF was added to harvest the total protein. Cells were harvested using a scraper. Subsequently, cell lysates were lysed for 30 min on ice followed by centrifugation for 10 min (12,000 rpm, 4°C), and the supernatants containing the total protein were collected. The Nuclear and Cytoplasmic Protein Extraction Kit (Beyotime Biotechnology) was used in accordance with the manufacturer’s instructions to extract the nuclear protein. The BCA Assay was used for protein quantification according to the manufacturer’s instructions (Thermo Fisher Scientific). First, 20 μg of protein samples were separated on an SDS-polyacrylamide gel and transferred to PVDF membranes (Millipore, Bedford, MA, USA). Then, the membranes were blocked with 5% bovine serum albumin for 1 h at room temperature. The membranes were then incubated overnight with primary antibodies for SOD1 (Cell Signaling Technology, Beverly, MA, USA), SOD2 (ProteinTech Group, Chicago, IL, USA), Nrf2 (ProteinTech Group), Keap1 (ProteinTech Group), NQO1 (ProteinTech Group), HO-1 (ProteinTech Group), aggrecan (ProteinTech Group), collagen II (ProteinTech Group), sex determining region Y-box 9 (SOX9) (Abcam, Cambridge, UK), matrix metalloproteinase 9 (MMP9, ProteinTech Group), matrix metalloproteinase 13 (MMP13, ProteinTech Group), collagen X (Abcam), inducible nitric oxide synthase (iNOS, ProteinTech Group), cyclooxygenase 2 (COX2, ProteinTech Group), GAPDH (Cell Signaling Technology), and lamin B1 (ProteinTech Group) at 4°C. Thereafter, the membranes were washed three times for 10 min each time and incubated using goat anti-rabbit or goat anti-mouse horseradish peroxidase (HRP)-labeled secondary antibody (Cell Signaling Technology) for 1 h at room temperature. Finally, the Immobilon Western Chemiluminescent HRP Substrate (Millipore) and the ImageQuant LAS 4000 mini system (Amersham Pharmacia GE, Boston, MA, USA) were used for detection and photography. Images were analyzed using Image J software [29].

### Statistical analysis

All statistical analyses were conducted with GraphPad Prism 9 for Mac (GraphPad Software Inc., San Diego, CA, USA). Numerical data are presented as means ± standard deviations. The independent two-tailed Student’s t-test was applied to compare results of two groups, and one-way analysis of variance was conducted to compare the variables between more than two groups. Statistical significance was defined as *p* < 0.05.

## Results

In the present study, the antioxidant and chondroprotective effects of EA on IL-1β-treated chondrocytes were assessed. First, the cytotoxicity of EA was examined to select optimal concentrations. Second, we found that EA could attenuate oxidative stress and exert protective effects on chondrocytes. Importantly, we found that EA could ameliorate oxidative stress and protect chondrocytes by upregulating the Keap1/Nrf2 signaling pathway.

### Effects of EA on cell viability of chondrocytes

The CCK8 assay was performed to evaluate the cytotoxic effects of EA on C28/I2 chondrocytes. In the absence of IL-1β (Figure 1(b)), EA at the doses of 10 and 25 μM displayed no cytotoxicity on chondrocytes at 24 h. However, cell viability decreased in the presence of 50 μM EA. In the presence of IL-1β (Figure 1(c)), EA at the concentrations of 10, 25, and 50 μM displayed no cytotoxicity on chondrocytes at 24 h. Nevertheless, cell viability decreased under treatment with 75 μM EA. Therefore, EA concentrations of 10 and 25 μM were used for subsequent experiments.

### EA attenuated IL-1β-induced oxidative stress and promoted cartilage matrix secretion of chondrocytes

**Figure 2.**
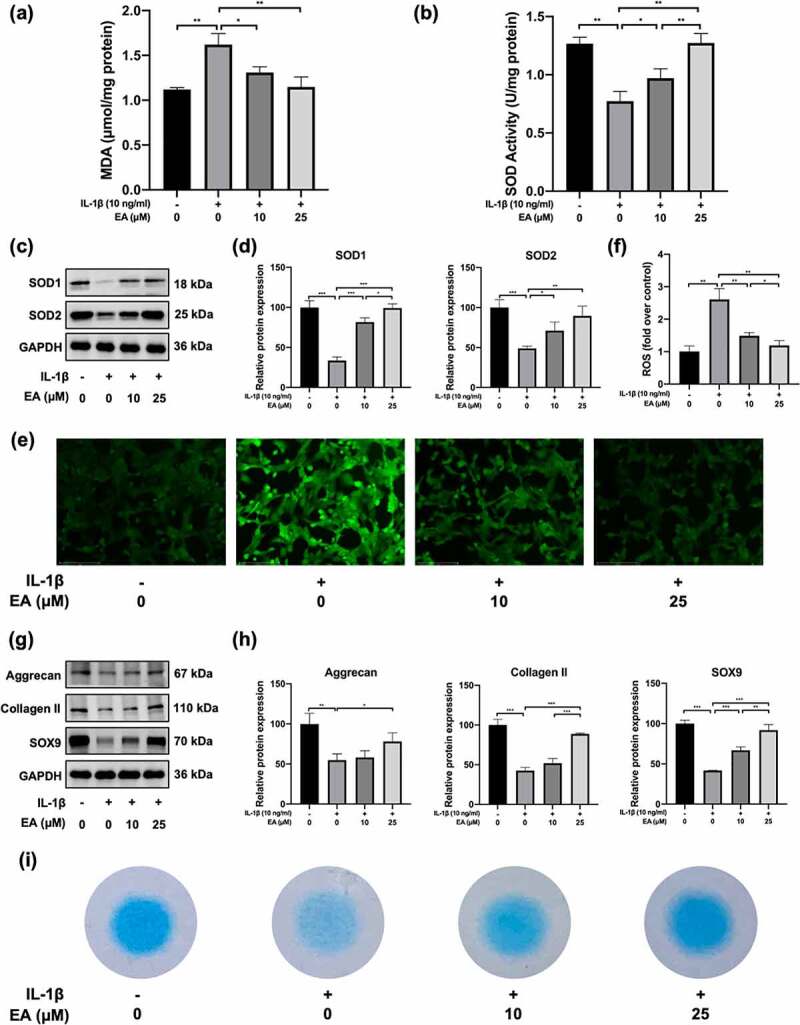
Ellagic acid (EA) attenuated oxidative stress and promoted cartilage matrix generation in interleukin (IL)-1β-exposed chondrocytes. (a) Malondialdehyde (MDA) levels were determined using the MDA assay. (b) Superoxide dismutase (SOD) activity levels were detected using the SOD activity assay. The protein expression of SOD1 and SOD2 was assessed by (c) western blot and (d) quantification analysis. Intercellular reactive oxygen species (ROS) levels were determined using (e) a 2,7-dichlorofluorescin diacetate (DCFH-DA) probe and (f) quantification analysis. The protein expressions of aggrecan, collagen II, and sex determining region Y-box 9 (SOX9) were detected by (g) western blot and (h) quantification analysis. (i) Secretion levels of sulfated proteoglycans were assessed using Alcian blue staining. *N* = 3. The values are expressed as the mean ± standard deviation. *p < 0.05, **p < 0.01, and ***p < 0.001.

To investigate the effects of EA on oxidative stress in chondrocytes, cells were treated with EA and IL-1β for 24 h. Indicators of oxidative stress, such as MDA levels, SOD activity, and intracellular ROS levels, as well as protein expression of antioxidases, including SOD1 and SOD2, were determined. As shown in Figure 2(a), IL-1β increased MDA levels, but EA treatment resisted the effects of IL-1β, which suggests that EA could attenuate the lipid oxidative and membrane lipid damage in chondrocytes under oxidative stress. Similarly, EA administration could reverse IL-1β-induced intracellular ROS levels (Figure 2(e, f)). In addition, EA treatment attenuated the IL-1β-induced suppression of SOD activity (Figure 2(b)), which was consistent with the increased protein expression of SOD1 and SOD2 (Figure 2(c, d)). These results indicate that EA attenuated oxidative stress in chondrocytes.

To detect the effects of EA on cartilage matrix generation in chondrocytes under IL-1β-induced oxidative stress, we evaluated the expression of aggrecan, collagen II, SOX9, and sulfated proteoglycans. As depicted in Figure 2(g, h), EA reversed the IL-1β-induced decrease in protein expression of aggrecan, collagen II, and SOX9. Similarly, EA inhibited the IL-1β-induced reduction of sulfated proteoglycan secretion in chondrocytes (Figure 2(i)). Aggrecan, collagen II, and sulfated proteoglycans are important ingredients of articular hyaline cartilage, and SOX is a transcription factor contributing to cartilage formation. These results suggest that administration of EA can promote cartilage matrix secretion in chondrocytes under oxidative stress.

### EA ameliorated chondrocytes injury under IL-1β-induced oxidative stress

**Figure 3.**
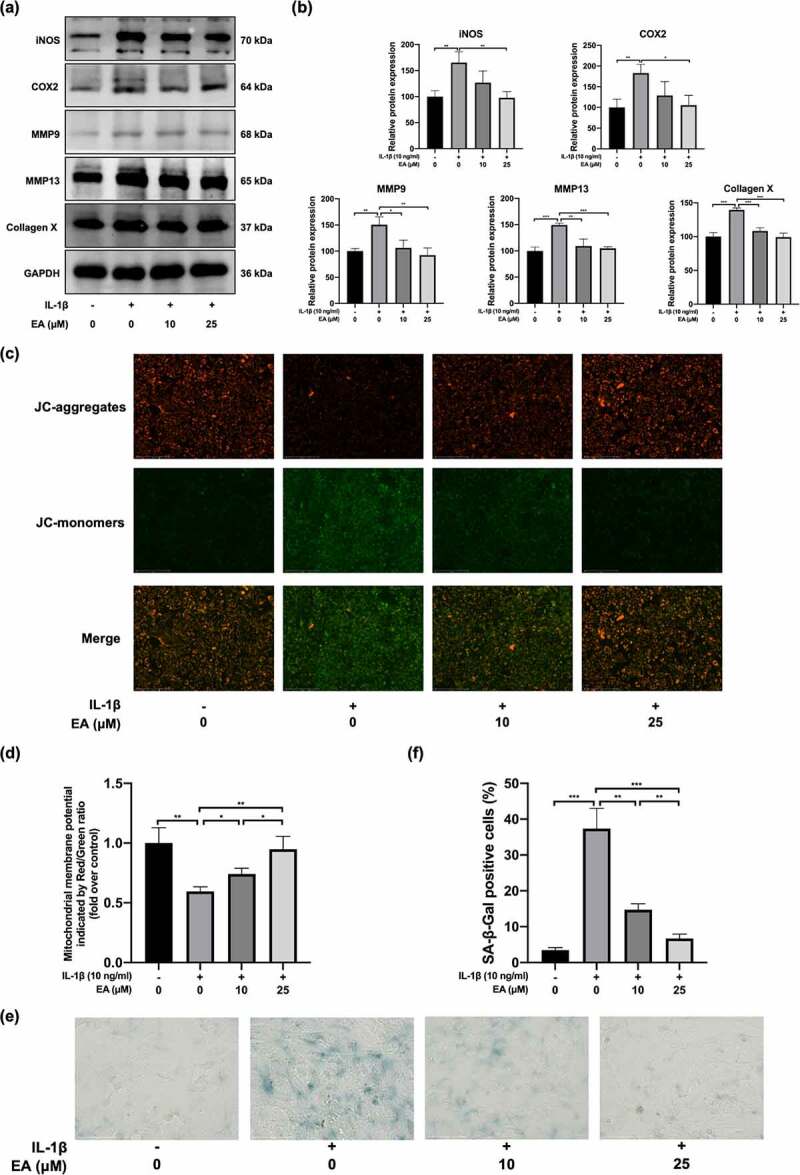
Ellagic acid (EA) ameliorated chondrocyte injury under interleukin (IL)-1β-induced oxidative stress. The protein expression of inducible nitric oxide synthase (iNOS), cyclooxygenase 2 (COX2), matrix metalloproteinase (MMP)9, MMP13, and collagen X were detected by (a) western blot and (b) quantification analysis. Mitochondrial membrane potential levels were assessed by (c) JC-1 staining and (d) quantification analysis. Cell senescence levels were assessed by (e) Senescence β-galactosidase (SA-β-Gal) staining and (f) quantification analysis. *N* = 3. The values are expressed as the mean ± standard deviation. *p < 0.05, **p < 0.01, and ***p < 0.001.

To assess the effects of EA on chondrocyte degradation under oxidative stress, we determined the cell injury degree according to the protein expression of proinflammatory cytokines (iNOS and COX2); a marker of chondrocyte hypertrophy, (collagen X); and catabolic enzymes, including MMP9 and MMP13. As shown in Figure 3(a, b), EA significantly suppressed the IL-1β-induced increase in COX2, iNOS, collagen X, MMP9, and MMP13 protein levels. Moreover, the detected mitochondrial membrane potential levels reflected the early apoptosis of cells. After EA administration, a significant elevation of the mitochondrial membrane potential was observed in IL-1β-exposed chondrocytes (Figure 3(c, d)), suggesting that EA can attenuate the apoptosis of chondrocytes under IL-1β-induced oxidative stress. In addition, EA delayed cell senescence of IL-1β-treated chondrocytes, as indicated by the results of SA-β-Gal staining (Figure 3(e, f)). Together, these results indicate that EA could ameliorate chondrocyte injury under oxidative stress.

### EA upregulated Keap1/Nrf2 signaling pathway in chondrocytes under IL-1β-induced oxidative stress

**Figure 4.**
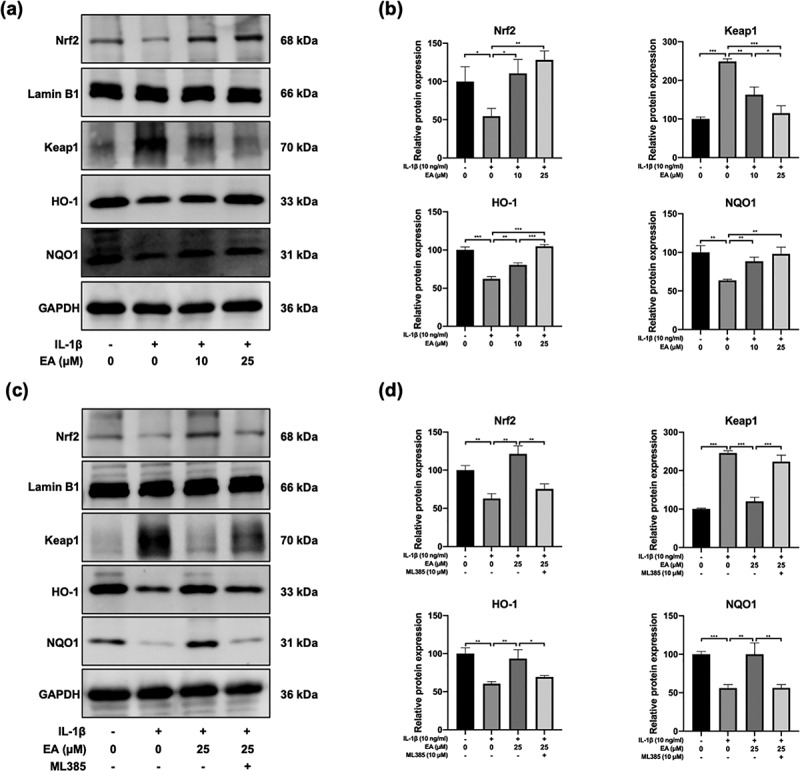
Ellagic acid (EA) upregulated the Keap1/Nrf2 signaling pathway in interleukin (IL)-1β-exposed chondrocytes. The protein expression of Nrf2, Keap1, heme oxygenase-1 (HO-1), and NADPH quinone oxidoreductase 1 (NQO1) in chondrocytes (a-b) treated with EA and IL-1β (10 ng/mL) or (c-d) exposed to EA, IL-1β (10 ng/mL), and a specific Keap1/Nrf2 pathway inhibitor (ML385, 10 μM) were detected by western blot and quantification analysis. *N* = 3. The values are expressed as the mean ± standard deviation. *p < 0.05, **p < 0.01, and ***p < 0.001.

Activation of the Keap1/Nrf2 signaling pathway plays a crucial role in antioxidant responses. In this study, western blotting was performed to determine the EA-regulated activation of Keap1/Nrf2 signaling pathway in chondrocytes under IL-1β-induced oxidative stress. As shown in Figure 4(a, b), IL-1β upregulated the expression of Keap1 but downregulated the expression of Nrf2 as well as the protein levels of downstream targets of Nrf2, HO-1, and NQO1. However, EA treatment reversed the IL-1β-induced increase in Keap1 and decreases in Nrf2, HO-1, and NQO1, as measured by changes in protein expression (Figure 4(a, b)). ML385, a specific Keap1/Nrf2 signal pathway inhibitor, was used to verify the effects of EA on the Keap1/Nrf2 signal pathway in chondrocytes. As shown in Figure 4(c, d), ML385 significantly inhibited the EA-induced upregulation of Nrf2, HO-1, and NQO1, and it reversed the EA-induced downregulation of Keap1 (Figure 4(c, d)). These results confirmed that EA can upregulate the Keap1/Nrf2 signaling pathway in chondrocytes under IL-1β-induced oxidative stress.

### EA attenuated IL-1β-induced oxidative stress and promoted cartilage matrix secretion of chondrocytes by upregulating the Keap1/Nrf2 signaling pathway

**Figure 5.**
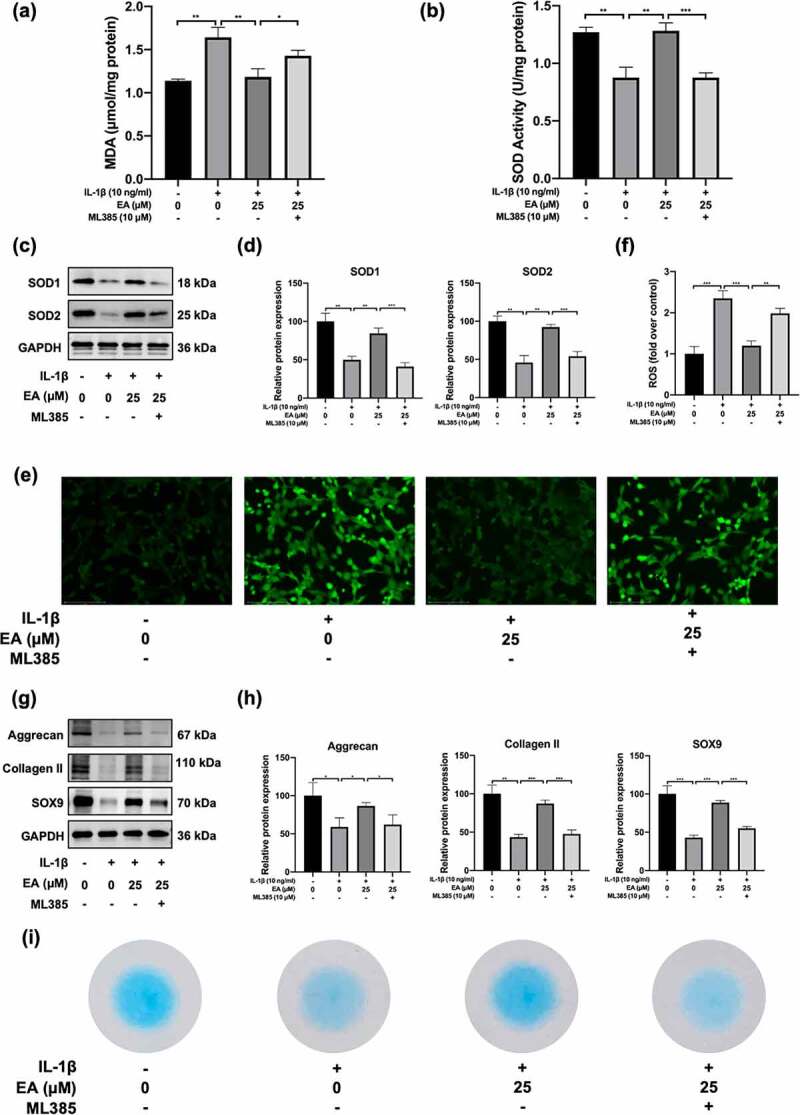
ML385 suppressed the antioxidant effects of ellagic acid (EA) and inhibited the EA-mediated increase in cartilage matrix secretion of interleukin (IL)-1β-exposed chondrocytes. C28/I2 chondrocytes were treated with Ellagic acid (EA), IL-1β (10 ng/mL), and ML385 (10 μM) for 24 h. (a) Malondialdehyde (MDA) levels were determined using the MDA assay. (b) Superoxide dismutase (SOD) activity levels were detected using the SOD activity assay. The protein expressions of SOD1 and SOD2 were assessed by (c) western blot and (d) quantification analysis. Intercellular ROS levels were determined using (e) a 2,7-dichlorofluorescin diacetate (DCFH-DA) probe and (f) quantification analysis. The protein expression of aggrecan, collagen II, and sex determining region Y-box 9 (SOX9) were determined by (g) western blot and (h) quantification analysis. (i) Secretion levels of sulfated proteoglycans were detected using Alcian blue staining. *N* = 3. The values are expressed as the mean ± standard deviation. *p < 0.05, **p < 0.01, and ***p < 0.001.

To determine whether the Keap1/Nrf2 signaling participated in the antioxidant effects of EA on chondrocytes under oxidative stress, we measured the ML385-induced variations in MDA and intracellular ROS levels as well as the total SOD activity and SOD1 and SOD2 protein expression of chondrocytes under IL-1β-induced oxidative stress and EA administration. We found that MDA and intracellular ROS increased significantly after ML385 treatment (Figure 5(a), Figure 5(e) and Figure 5(f)), indicating that ML385-induced downregulation of the Keap1/Nrf2 signaling pathway suppressed the antioxidant effects of EA. In addition, total SOD activity and SOD1 and SOD2 protein expression decreased significantly after ML385 treatment (Figure 5(b–d)). These results indicated that EA attenuated oxidative stress in chondrocytes via upregulation of the Keap1/Nrf2 signaling pathway.

To verify whether EA restored cartilage matrix secretion of chondrocytes under IL-1β-induced oxidative stress via the Keap1/Nrf2 signaling pathway, we examined ML385-induced changes in expression of aggrecan, collagen II, SOX9, and sulfated proteoglycans of chondrocytes under IL-1β-induced oxidative stress and EA administration. Western blotting analysis indicated that ML385 treatment decreased the expression of aggrecan, collagen II, and SOX9 (Figure 5(g) and Figure 5(h)). Meanwhile, ML385 downregulated the EA-mediated sulfated proteoglycan generation recovery of chondrocytes subjected to IL-1β-induced oxidative stress (Figure 5(i)). These results indicate that EA restores the cartilage matrix secretion functions in chondrocytes by upregulating the Keap1/Nrf2 signaling pathway.

### EA ameliorated chondrocyte injury under IL-1β-induced oxidative stress by upregulating the Keap1/Nrf2 pathway

**Figure 6.**
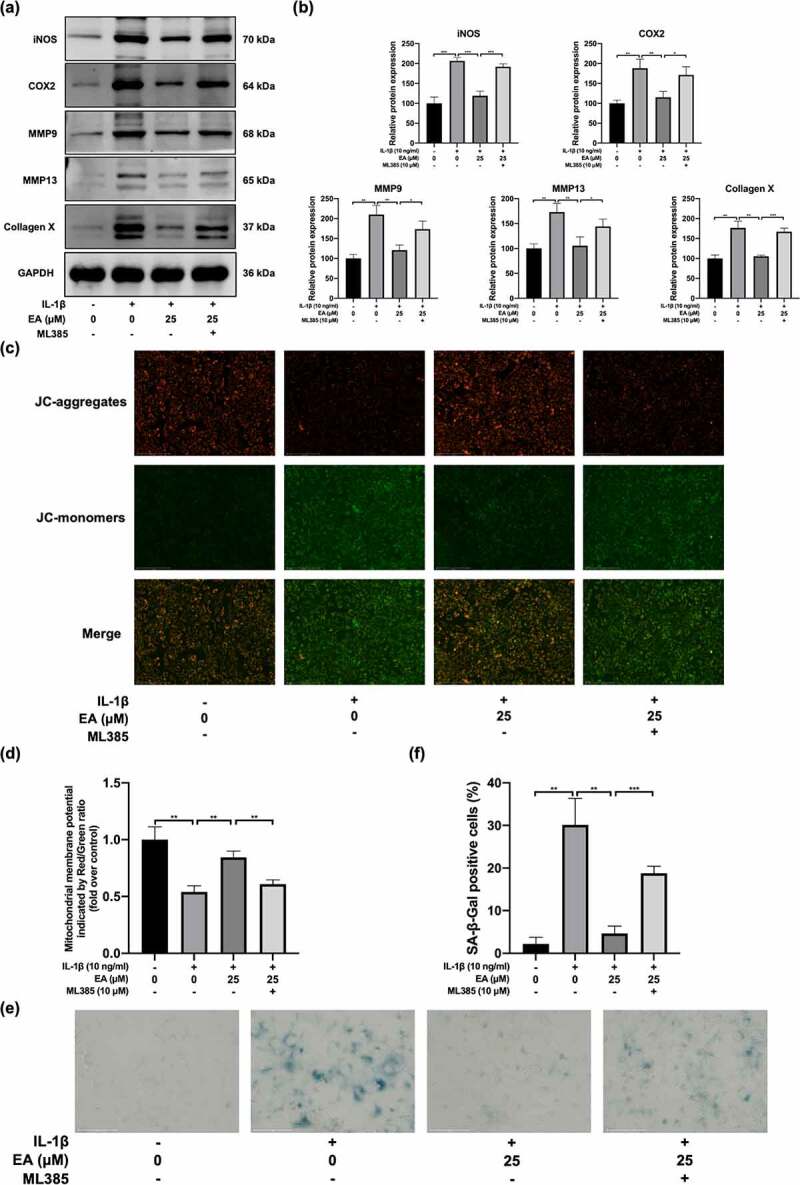
ML385 reversed the ellagic acid (EA)-mediated remission of the chondrocyte injury subjected to interleukin (IL)-1β-induced oxidative stress. C28/I2 chondrocytes were treated with ellagic acid (EA), IL-1β (10 ng/mL), and ML385 (10 μM) for 24 h. The protein expression levels of inducible nitric oxide synthase (iNOS), cyclooxygenase 2 (COX2), matrix metalloproteinase (MMP)9, MMP13, and collagen X were detected by (a) western blot and (b) quantification analysis. Mitochondrial membrane potential levels were detected by (c) JC-1 staining and (d) quantification analysis. Cell senescence levels were assessed by (e) senescence β-galactosidase (SA-β-Gal) staining and (f) quantification analysis. *N* = 3. The values are expressed as the mean ± standard deviation. *p < 0.05, **p < 0.01, and ***p < 0.001.

To determine whether EA ameliorated chondrocyte injury under IL-1β-induced oxidative stress through Keap1/Nrf2 signaling pathway, we evaluated the ML385-mediated variation of indicators of chondrocyte degradation, including mitochondrial membrane potential levels, cellular senescence, and protein expression of iNOS, COX2, MMP9, MMP13, and collagen X. As shown in Figure 6(a) and Figure 6(b), ML385 treatment reversed the EA-mediated downregulation of iNOS, COX2, MMP9, MMP13, and collagen X, as reflected by measurements of protein levels. In addition, EA-mediated reduction of the mitochondrial membrane potential was weakened by ML385 treatment (Figure 6(c) and Figure 6(d)). ML385 treatment also suppressed the attenuation of EA effects on chondrocyte senescence caused by IL-1β-induced oxidative stress (Figure 6(e) and Figure 6(f)). These results suggest that EA attenuates chondrocyte degradation by upregulating the Keap1/Nrf2 signaling pathway. This data indicate that EA attenuates chondrocyte injury under IL-1β-induced oxidative stress by upregulating the Keap1/Nrf2 signaling pathway.

## Discussion

OA is the most prevalent joint disease in older adults; it causes pain and joint dysfunction and ultimately reduces quality of life [30]. Current therapies for OA, such as hyaluronic acid, opioids, and corticosteroids, focus primarily on relief of symptoms, but do not reverse the progression of OA [31–33]. Effective nonoperative strategies that could delay the pathological process of OA are urgently needed. Herein, we investigated the underlying mechanisms of the therapeutic effects of EA using C28/I2 normal human chondrocytes exposed to IL-1β. Our results indicate that EA effectively protects chondrocytes from oxidative stress, dysfunction, and degradation by upregulating the Keap1/Nrf2 signaling pathway.

In recent years, the effects of oxidative stress on OA have been identified [12]. Oxidative stress is associated with articular cartilage degeneration and the development of OA [34]. Overproduction of ROS increases the susceptibility of chondrocytes to oxidant-mediated cell death and reduces antioxidant functions [35]; thus, natural antioxidants might be a useful approach to OA management. A recent study reported that natural antioxidants, including allicin, lycopene, sulforaphane, and N-acetyl-L-cysteine (NAC), attenuated oxidative stress and exerted protective effects on human osteoarthritic chondrocytes through the Keap1/Nrf2 signaling pathway [36]. EA, a natural phenolic compound with four hydroxyl (H bond acceptor) and two lactone (H bond donor) functional groups [37], abundantly exists in plant extracts [38]; it possesses various pharmacological properties, including cardioprotective, anti-inflammatory, and antioxidant effects [39]. In this study, we used IL-1β to induce oxidative stress and degrade chondrocytes, as indicated by increases in MDA and intracellular ROS levels as well as upregulation of protein expression of iNOS, COX2, MMP9, MMP13, and collagen X. However, all these variations could be restored by EA administration. Moreover, we found that EA attenuated the IL-1β-induced early apoptosis and senescence of chondrocytes. Previous studies have demonstrated that oxidative stress with an increase in ROS level is critical for the induction and maintenance of cell senescence processes [40]. Additionally, treatment with EA reversed the IL-1β-induced decrease in the expression of aggrecan, collagen II, SOX9 and sulfated proteoglycans of chondrocytes, suggesting that EA could alleviate chondrocyte dysfunction. These findings demonstrated that EA could protect chondrocytes from IL-1β-induced-oxidative stress and exerts chondroprotective effects.

To detect the underlying mechanism of EA alleviation of oxidative stress in chondrocytes, we examined the expression of Nrf2 and Keap1 protein. Nrf2 is an oxidative stress-sensitive transcription factor and serves as a crucial regulator of cellular redox homeostasis [13]. In addition, activation of the Nrf2/ARE pathway exerts antioxidative effects in OA chondrocytes [41,42]. Moreover, Hesu et al. demonstrated that EA could significantly increase the expression of antioxidant enzymes, such as SOD and HO-1, through upregulation of Nrf2 and could noticeably weaken the cytoplasmic stability of Keap1 [43]. Therefore, we speculated that EA might ameliorate oxidative stress-induced dysfunction in chondrocytes through the Keap1/Nrf2 signaling pathway. In the present study, we alleviated oxidative stress and dysfunction in IL-1β-treated human chondrocytes using EA and confirmed that the Keap1/Nrf2 signaling pathway was involved in the progression. IL-1β-treated chondrocytes expressed more Keap1 and less Nrf2 compared to the control group. Furthermore, the protein levels of the downstream targets of Nrf2 (HO-1 and NQO1) decreased after IL-1β intervention. Our results indicated that EA reversed all these changes, which suggested that EA could upregulated the Keap1/Nrf2 pathway in chondrocytes under oxidative stress. We also used ML385 (10 μM), a specific Keap1/Nrf2 signaling pathway inhibitor, to detect the Keap1/Nrf2 pathway involvement in the antioxidant and chondroprotective abilities of EA. We found that ML385 downregulated the protein expression of Nrf2, NQO1, and HO-1. ML385 administration suppressed the antioxidant and protective effects of EA on chondrocytes subjected to IL-1β-induced oxidative stress by upregulating the Keap1/Nrf2 pathway.

In this study, we determined that EA could exert antioxidant and chondroprotective effects on chondrocytes by upregulating the Keap1/Nrf2 signaling pathway. However, this study has some limitations that should also be considered. The therapeutic effects of EA on OA and the underlying mechanisms must be investigated *in vivo*. In addition, our study was limited to IL-1β stimulation, and the use of EA as an OA treatment should be confirmed by future *in vitro* and *in vivo* experiments. Despite the shortcomings of this study, it was the first, to our knowledge, to demonstrate that EA could ameliorate oxidative stress in IL-1β-treated chondrocytes and exert chondroprotective effects through the Keap1/Nrf2 pathway, which suggested that EA was a promising bioactive agent applied to functionalize biomaterials for cartilage defect regeneration in the OA articular cavity with oxidative stress.

## Conclusion

This study demonstrated that EA attenuated IL-1β-induced oxidative stress and exerted chondroprotective effects on chondrocytes through up-regulation of the Keap1/Nrf2 signaling pathway. These findings provide a novel perspective on the therapeutic effects of EA on OA, and suggest that EA was a promising antioxidant applied to therapy for degenerative diseases.

## Data Availability

The original contributions presented in the study are included in the article, further inquiries can be directed to the corresponding author.
